# Decomposition of musculoskeletal structures from radiographs using an improved CycleGAN framework

**DOI:** 10.1038/s41598-023-35075-x

**Published:** 2023-05-25

**Authors:** Naoki Nakanishi, Yoshito Otake, Yuta Hiasa, Yi Gu, Keisuke Uemura, Masaki Takao, Nobuhiko Sugano, Yoshinobu Sato

**Affiliations:** 1 Division of Information Science, Graduate School of Science and Technology, Nara Institute of Science and Technology, Ikoma, Nara 630-0192 Japan; 2 Department of Orthopaedic Medical Engineering, Osaka University Graduate School of Medicine, Suita, Osaka 565-0871 Japan; 3 Department of Bone and Joint Surgery, Ehime University Graduate School of Medicine, Toon, Ehime 791-0295 Japan

**Keywords:** Radiography, Three-dimensional imaging

## Abstract

This paper presents methods of decomposition of musculoskeletal structures from radiographs into multiple individual muscle and bone structures. While existing solutions require dual-energy scan for the training dataset and are mainly applied to structures with high-intensity contrast, such as bones, we focused on multiple superimposed muscles with subtle contrast in addition to bones. The decomposition problem is formulated as an image translation problem between (1) a real X-ray image and (2) multiple digitally reconstructed radiographs, each of which contains a single muscle or bone structure, and solved using unpaired training based on the CycleGAN framework. The training dataset was created via automatic computed tomography (CT) segmentation of muscle/bone regions and virtually projecting them with geometric parameters similar to the real X-ray images. Two additional features were incorporated into the CycleGAN framework to achieve a high-resolution and accurate decomposition: hierarchical learning and reconstruction loss with the gradient correlation similarity metric. Furthermore, we introduced a new diagnostic metric for muscle asymmetry directly measured from a plain X-ray image to validate the proposed method. Our simulation and real-image experiments using real X-ray and CT images of 475 patients with hip diseases suggested that each additional feature significantly enhanced the decomposition accuracy. The experiments also evaluated the accuracy of muscle volume ratio measurement, which suggested a potential application to muscle asymmetry assessment from an X-ray image for diagnostic and therapeutic assistance. The improved CycleGAN framework can be applied for investigating the decomposition of musculoskeletal structures from single radiographs.

## Introduction

Quantitative assessments and patient-specific simulations of musculoskeletal systems have been studied extensively in orthopedic surgeries for effective treatment planning and investigation of disease progression. The evaluation of the volume and density of each individual muscle is useful in the diagnosis of muscular dystrophy and sarcopenia^1–3^, rehabilitation planning^4,5^, and personalized biomechanics simulation^6,7^. However, the challenge in the existing method is difficulty in the measurement of muscle mass on a regular basis because three-dimensional (3D) imaging, such as CT and MRI, imposes a high burden on the patient due to radiation exposure in CT, long scan time in MRI, and high cost in both CT and MRI.

Radiography exposes patients to far less radiation than CT, making it a more useful modality in routine clinical settings than both CT and MRI. It is thus used for routine clinical checkups of skeletal alignment or joint disease progression, such as knee osteoarthritis^8^. However, the analyses in radiography have been limited to bones. A method for bone decomposition from a radiography image, that is, separation of intensity components due to bones from those due to soft tissues, was developed, and its clinical importance was demonstrated by a significant improvement in bone mineral density estimation accuracy compared with estimation from a non-decomposed X-ray image^9^. However, for training, this method used paired and accurately registered datasets of X-ray images and CT data, which is not only difficult in training data construction but also difficult for the application to muscles because the muscles deform even between radiography and CT imaging. Radiography decomposition into individual muscles and bones using unpaired training data could become a low-cost, low-radiation-dose alternative for frequent monitoring of detailed musculoskeletal conditions, including muscle mass measurement. We are not aware of any method that allows for the decomposition and measurement of muscle mass from a radiography image.

**Figure 1:**
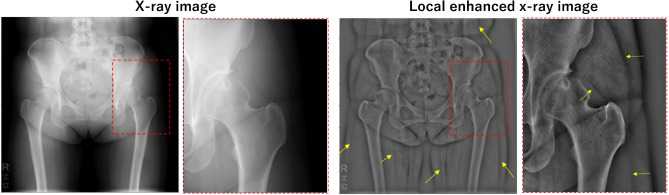
Muscle edges observed in a contrast-enhanced X-ray image. The X-ray image and its contrast-enhanced image^10,11^ are shown on the left and right, respectively. The lower panels show magnified view. The yellow arrows indicate the muscle edges confirmed by an expert surgeon.

One motivating evidence toward addressing muscles in X-ray images in this study is that the edges of some muscles are partly visible to human eyes in local contrast-enhanced X-ray images^10,11^ (Fig. 1). We hypothesized that these X-ray images contain information sufficient for discriminating individual muscles in addition to bones. This study aimed to decompose individual muscle and bone regions from an X-ray image. We formulated the problem as an image translation from a real X-ray image to a set of decomposed images of individual muscles and bones, and we solved it using generative adversarial networks (GANs)^12^. As a training dataset, we used muscle and bone regions in CT automatically segmented using Bayesian U-net^6^ and generated digitally reconstructed radiographs (DRRs) of each muscle or bone combined with real X-ray images.

### Related work

Most related works can be classified into segmentation and decomposition as summarized in Table 1. The pixel-wise classification is called *segmentation*, whereas the pixel-wise estimation of multiple continuous attenuation values of individual structures that compose each X-ray’s line integral (i.e., the sum of X-ray attenuation along one X-ray beam) is called *decomposition*.

#### Segmentation

Novikov et al.^13^, Chen et al.^14^, and Eslami et al.^15^ proposed deep learning-based segmentation methods for one of (or all of) the lung, heart, and clavicle regions. They used manually annotated label images of the target regions on the X-ray images, which incur a high annotation cost. Zhang et al.^16,17^ employed DRRs created from CT images.

Their method is advantageous because the diverse training dataset could be automatically generated by perturbing translation and rotation of the CT. However, the integration of attenuation was not considered in the above studies; thus, decomposition was not realized.

#### Decomposition

One classical machine learning approach for X-ray image decomposition used the training data set acquired using dual-energy X-ray absorptiometry (DXA). Suzuki et al.^18^ and Yang et al.^19^ performed bone suppression (i.e., bone tissue decomposition) from chest X-ray images. While the focus in these studies was enhancing lung lesions covered by rib bones, the same approach does not apply to the decomposition of multiple soft tissues due to the limitation of discriminative ability in DXA acquisition. Li et al.^20,21^ proposed an X-ray image decomposition method for bone suppression by converting chest X-ray images into DRRs of bone and lung regions. However, quantitative evaluation of the decomposed DRRs with the real X-ray images has not been performed. In addition to bones, Albarqouni et al.^22^ performed the decomposition of multiple organs from X-ray images. The decomposition model was trained by pairing X-ray image-like DRRs created by projecting CT images as inputs and a series of decomposed DRRs created using CT images and mask images as outputs. They introduced a new loss term, reconstruction loss, which considers that multiple regions are superimposed to “reconstruct” the original X-ray image. The proposed reconstruction loss was tested only on DRRs generated from calibrated CT images in an ideal condition; the method was not evaluated on real X-ray images. Gu et al.^9^ recently proposed a method for quantitatively decomposing a partial bone region, namely, the proximal femur region, from an X-ray image to measure bone mineral density. Howerver, this work was also limited to bones.

To the best of our knowledge, no study has considered the following two features, which are essential for real clinical applications in musculoskeletal diagnosis.

- Decomposition of multiple soft tissues (i.e., muscles) in addition to bones
- Quantitative evaluations of decompositions from real X-ray images

Image translation using GANs^12^ has been extensively studied and shown to outperform conventional methods. However, in our context of muscle tissue decomposition from X-ray image using DRRs created from CT, obtaining a paired training dataset (i.e., input and output images are registered to each other) was difficult due to the shape changes (even slightly) of flexible muscles in different poses between CT and X-ray image scans. Therefore, algorithms that can handle unpaired training data, such as cycle-consistent GAN (CycleGAN)^23^, are our focus. Several recent works^24,25^ proposed an improved version of CycleGAN and compared the performance using the original CycleGAN as the baseline. Hiasa et al.^26^ introduced a new loss term in CycleGAN, the gradient correlation (GC) loss, which preserves the alignment of edges by calculating the correlation of gradients in input and output images. Wang et al.^27^ proposed an improvement of the Pix2Pix model^28^, a commonly employed GAN model for image translation that uses a paired training dataset by incorporating hierarchical learning (HL) for high-resolution image translation.

In this study, we integrated the three previously proposed features, i.e., the reconstruction loss^22^, GC loss^26^, and HL^27^, into the CycleGAN framework to achieve edge-alignment preserved and high-resolution image decomposition of real X-ray images.

The contribution of this study is three-fold. (1) Proposal of methods to decompose multiple soft tissues (i.e., muscles) and bones from real X-ray images. (2) Integration of the three previously proposed features in different contexts into CycleGAN to achieve high-resolution stable image translation robust against intensity variation in real X-ray images. (3) Thorough evaluations of the proposed methods using a dataset comprising real X-ray images and CT images of 475 patients, including evaluations with a metric on muscle asymmetry.

**Table 1:** Comparison with previous studies.

Study	Task	Training dataset	Technique	Target anatomy
Proposed	Decomposition	Automatically annotated CT	Cycle GAN	Bone, muscle
Novikov et al.^13^	Segmentation	Manually annotated X-ray	U-net	Lung, clavicle, heart
Chen et al.^14^	Segmentation	Manually annotated X-ray	GAN	Lung
Eslami et al.^15^	Segmentation and bone suppression	Manually annotated X-ray	Conditional GAN	Lung, heart
Zhang et al.^16^	Segmentation	Manually annotated CT	GAN	Lung, heart, liver, bone
Suzuki et al.^18^	Bone suppression	Dual-energy X-ray	Neural Network	Lung
Yang et al.^19^	Bone suppression	Dual-energy X-ray	CNN	Lung
Li et al.^21^	Bone suppression	Manually annotated CT	Cycle GAN	Lung
Albarqouni et al.^22^	Decomposition of organs	Manually annotated CT	U-net	Rib cage, vessel, spine, thorax
Gu et al.^9^	Decomposition of bone	Automatically annotated CT	GAN	Proximal femur

## Methods

**Figure 2:**
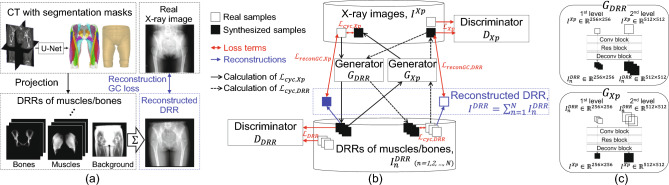
Proposed method. (**a**) Preparation of training datasets. Digitally reconstructed radiographs (DRRs) of the individual muscle/bone regions are created using CT images and their corresponding 3D masks automatically generated by a pretrained Bayesian U-net. The sum of the DRRs, which we call a reconstructed DRR, is used to calculate the reconstruction GC loss. (**b**) Data and process workflow. The CycleGAN framework comprising $G_{DRR}$, $G_{Xp}$, $D_{DRR}$ and $D_{Xp}$ was extended by adding the reconstruction GC loss, which suppresses the edge misalignment. (**c**) Hierarchical learning of generators, where the generators $G_{DRR}$ and $G_{Xp}$ are trained by the low-resolution and high-resolution images successively.

The overview of the methods is shown in Fig. 2. The training dataset comprises real X-ray images and their associated DRRs. The DRRs of individual muscle and bone regions are created using CT images and their associated 3D muscle/bone masks, which are automatically segmented from CT^6^. In the following subsections, we describe the projection model used in this study, proposed methods for the decomposition of X-ray images, implementation details, and evaluation metrics.

### Projection model

Our projection model assumes a point X-ray source (i.e., pinhole camera model) and one virtual X-ray beam per detector element, which ignores the effect of the finite focal spot size and finite area of the detector pixel. Furthermore, the pixel intensity at two-dimensional (2D) position $\textbf{u}$ $= (u, v)$ in an X-ray image $I^{X_p}\mathbf (u)$ is approximated as a line integral of a linear attenuation coefficient (AC) derived from a CT value along a ray penetrating an object, where the scattering and beam hardening effects are ignored. We consider that the abovementioned assumptions are reasonable to well-approximate a virtual X-ray image in light of many previous studies.

We assume that an X-ray image $I^{X_p}(\textbf{u})$ is regarded as the superimposition of the virtual projection (DRR) images $I^{DRR}_{n}(\textbf{u})$ of *K* individual muscle/bone regions $c_n$ ($1 \le n \le K$) and the background, i.e., the regions other than the *K* regions (e.g., fat, pelvic viscera, and vessels), denoted as $c_{K+1}$.

Thus, $I^{X_p}(\textbf{u})$ is approximated by

1 $$\begin{aligned} I^{X_p}(\textbf{u})&\approx \int _{0}^{d} \mu (s; {\textbf {u}}) ds = \sum _{n=1}^{K+1} \int _{0}^{d} \delta _n(s;{\textbf {u}}) \mu (s;{\textbf {u}}) ds = \sum _{n=1}^{K+1} I^{DRR}_{n}({\textbf {u}}), \end{aligned}$$

where *d* denotes the distance between the X-ray source and detector, $\mu (s;\textbf{u})$ denotes the one-dimensional (1D) profile of the AC along a ray penetrating the CT image, which corresponds to the pixel position ${\textbf {u}}$ on the 2D projection plane, *s* is an arc length parameter on the ray, and $\delta _n(s;\textbf{u})$ is the 1D binary profile of the segmentation mask along the ray corresponding to ${\textbf {u}}$ of the *n*-th muscle/bone region in the CT image.

The segmentation masks were automatically obtained from CT images using Bayesian U-net^6^. We used the distance between the source and the detector recorded in the DICOM header of the X-ray image (the attribute named “Distance Source to Detector”) for generating DRRs. To obtain an approximate patient position with respect to the X-ray imager coordinate system, each CT image was registered to the X-ray image of the same patient using a rigid 2D–3D registration algorithm proposed in^29^. Notably, owing to the hip angle difference and deformation of muscles between a CT image (acquired in supine position) and an X-ray image (acquired in standing position), the rigid registration only roughly aligned overall structures, which is the reason we required an unpaired image translation algorithm, we explain next.

### Hierarchical CycleGAN and reconstruction GC loss

For an unpaired image translation from a real X-ray image $I^{Xp}$ to a set of virtual projection images of the individual muscle/bone regions $I^{DRR}_{n}$, the CycleGAN proposed by Zhu et al.^23^ was employed. Figure 2b shows the workflow of the proposed image translation. The generators $G_{DRR}$ and $G_{Xp}$, generate $I^{DRR}_{n}$ from $I^{Xp}$ and $I^{Xp}$ from $I^{DRR}_{n}$, respectively. The discriminators $D_{Xp}$ and $D_{DRR}$ discriminate whether the image is real or fake.

The adversarial loss, $\mathcalligra {L}_{GAN}$, that we used in this study is defined as follows:

2 $$\begin{aligned} \mathcalligra {L}_{GAN}(G_{Xp},G_{DRR},D_{Xp},D_{DRR}) = \mathcalligra {L}_{Xp}(G_{Xp}, D_{Xp}) + \mathcalligra {L}_{DRR}(G_{DRR}, D_{DRR}), \end{aligned}$$

where

$$\begin{aligned} \mathcalligra {L}_{Xp}(G_{Xp},D_{Xp})&= \sum _{x \in I^{Xp}} \log D_{Xp}(x) + \sum _{y \in I^{DRR}} \log (1-D_{Xp} (G_{Xp}(y))) \\ \mathcalligra {L}_{DRR}(G_{DRR},D_{DRR})&= \sum _{y \in I^{DRR}} \log D_{DRR}(y) + \sum _{x \in I^{Xp}} \log (1-D_{DRR} (G_{DRR}(x))), \end{aligned}$$

in which *x* and *y* are images from domains $I^{X_p}$ and $I^{DRR}_n$, respectively. The cycle consistency loss $\mathcalligra {L}_{cyc}$ is given by

3 $$\begin{aligned} \mathcalligra {L}_{cyc}(G_{Xp},G_{DRR})&= \mathcalligra {L}_{cyc,Xp} + \mathcalligra {L}_{cyc,DRR} = \sum _{x \in I^{Xp}} |G_{Xp}(G_{DRR}(x)) - x |+ \sum _{y \in I^{DRR}} |G_{DRR}(G_{Xp}(y)) - y |. \end{aligned}$$

One challenge in the generation of a high-resolution image such as an X-ray image is to ensure stability. Because a higher resolution makes it easier to differentiate generated and training images, the discriminator converges faster than the generator, making the training unstable^30^. Therefore, we incorporate an approach using multiple resolution training images which is inspired by HL proposed for paired image translation^27^ into our unpaired image translation. The network structure is shown in Fig. 2c. The weight parameters for the generators, $G_{DRR}$ and $G_{Xp}$, are trained with the low-resolution images first and subsequently with the high-resolution images.

We employ a loss term that we call reconstruction GC loss to match the gradient in the sum of the decomposed images with the gradient of the input X-ray image. Albarqouni et al.^22^ defined the reconstruction loss term as the $\ell _2$-loss between the sum of decomposed images and input images. Although $\ell _2$-loss works in a simulation experiment as in^22^ where the input image was also DRRs generated from CT image, it will not be straightforward to apply the same loss term directly to real X-ray images because of the discrepancy from the ideal projection due to various physical phenomena, including the variation in scan protocol (e.g., X-ray tube current, and voltage), beam hardening, scattering, blurring due to focal spot size, and postprocessing filters built in the imaging system (e.g., bone enhancement filter). To improve robustness against the variation in absolute intensity, we incorporate gradient-matching constraints proposed in^26^, which use the GC^31^ in the regularization term.

The GC between the input image $I^{Xp}$ and $I^{DRR}$ reconstructed by summation of each decomposed image $\sum _n I_n^{DRR}$ is used in the regularization term, which we named the reconstruction GC loss and defined as follows:

4 $$\begin{aligned} \mathcalligra {L}_{reconGC}(G_{Xp},G_{DRR})&=\mathcalligra {L}_{reconGC,Xp}+\mathcalligra {L}_{reconGC,DRR} \nonumber \\&= \frac{1}{2} \left\{ \sum _{x \in I^{Xp}}\left( 1 - GC \left( x, \underbrace{\sum _n G_{DRR,n}(x)}_{\text {reconstruction}} \right) \right) \right. \left. + \sum _{y \in I^{DRR}}\left( 1 - GC \left( \underbrace{\sum _n y_n}_{\text {reconstruction}}, G_{Xp}(y)\right) \right) \right\} , \end{aligned}$$

where $\sum _n G_{DRR,n}(x)$ represents the sum of the multichannel output of the generator $G_{DRR}$, $y \in I^{DRR}$ represents one channel from the multichannel DRR. *GC*(*A*, *B*) is given by

5 $$\begin{aligned} GC(A,B) = \frac{1}{2} \{ NCC(\nabla _u A, \nabla _v B) + NCC(\nabla _u A, \nabla _v B) \} \end{aligned}$$

where *NCC*(*A*, *B*) denotes the normalized cross-correlation of *A* and *B*, and $\nabla _u$ and $\nabla _v$ are, respectively, *u* and *v* components of the gradient vector.

Finally, the total loss function, $L_{total}$, is formulated as follows:

6 $$\begin{aligned}&\mathcalligra {L}_{total}(G_{Xp},G_{DRR},D_{Xp},D_{DRR}) = \mathcalligra {L}_{GAN}(G_{Xp},G_{DRR},D_{Xp},D_{DRR}) + \lambda _{cyc} \mathcalligra {L}_{cyc}(G_{Xp},G_{DRR})\nonumber \\&\quad + \lambda _{GC} \mathcalligra {L}_{reconGC}(G_{Xp},G_{DRR}), \end{aligned}$$

where $\lambda _{cyc}$ and $\lambda _{GC}$ are the hyperparameters determining the balance among the three terms.

### Implementation details

The hierarchical CycleGAN incorporating the reconstruction GC loss was trained by solving the mini-max problem, given by

7 $$\begin{aligned} \hat{G}_{Xp}, \hat{G}_{DRR} = \textrm{arg} \min _{ {G_{Xp}, G_{DRR}}}\max _{ {D_{Xp},D_{DRR}}} \mathcalligra {L}_{ {total}} {(G_{Xp},G_{DRR},D_{Xp},D_{DRR})} \end{aligned}$$

using Adam^32^ with a learning rate of 0.0002 for the first 100 epochs and linearly decreasing to 0 for the later 100 epochs.

The generator was trained hierarchically with nine residual blocks, and the discriminator was $142 \times 142$ PatchGAN^33^. The balance parameters were experimentally determined to be $\lambda _{cyc}=10$ and $\lambda _{GC}=1.0$.

The average training time for the conventional and proposed methods was approximately 8.0 and 9.7 h, respectively (2.7 and 7.0 h for the training of global ($G_1$) and local ($G_2$) mappings, respectively), on a workstation equipped with an Intel Xeon processor (2.30 GHz, 4 cores) with NVIDIA Titan RTX (24 GB memory). The average computation time for the inference for one case was about 2.5 s, excluding the file access.

### Muscle asymmetry metric

We propose the volume ratio of left and right muscles as an evaluation metric, which will be potentially used as a diagnostic metric to evaluate muscle asymmetry directly measured from a plain X-ray image.

As described later in “Data set”, the patients of the dataset used in the experiments were before undergoing total hip arthroplasty, and one side, the operation side, was more affected than the other side. Therefore, the ratio of the affected/unaffected volumes, $V^{A}_n$/$V^{U}_n$ of muscle $c_n$, denoted by $r^{A:U}_n$, is used for the evaluation.

Our measurement of the volume ratio using a single 2D projection image is based on a parallel projection model (i.e., all rays penetrate the volume in parallel). The sum of line integrals within the muscle region $\Omega$ of an affected or unaffected side in a 2D DRR is given by

8 $$\begin{aligned} \int _\Omega I^{DRR} d\Omega = \int _\Omega \int _0^d \delta (s)\mu (s) ds d\Omega = \alpha \int ^{d}_{0} \delta (s) ds = \alpha V \end{aligned}$$

where *V* denotes the 3D volume of the affected or unaffected side of the target muscle and $\alpha$ denotes the average AC within its region. Equation (8) indicates that the sum of the intensity value in $I^{DRR}$ can be approximated as a constant multiple of volume *V* of each muscle $\alpha V$. Therefore, the ratio of the volumes, in particular, AC-weighted volumes, of muscles between the affected and unaffected sides, $r^{A:U}$ (hereinafter “affected/unaffected volume ratio”), can be estimated only from the decomposed DRR images by

9 $$\begin{aligned} r^{A:U} = \frac{\int _\Omega I^{DRR,A}d\Omega }{\int _\Omega I^{DRR,U}d\Omega }\approx \frac{\alpha ^A V^A}{\alpha ^U V^U} \end{aligned}$$

where $I^{DRR,A}$ and $\alpha ^A$ are the DRR and average AC of the affected side, respectively, and $I^{DRR,U}$ and $\alpha ^U$ are those of the unaffected side, respectively. Its accuracy evaluation is demonstrated later in “Real image experiments” by comparison with the volume ratio obtained from the 3D mask of each muscle, which was automatically segmented from CT. According to^34^, around 20% decrease in the volume ratio and around 10 HU decrease in the CT value difference occur on average between the unaffected and affected sides. The 10 HU decrease causes around 1% decrease in the average AC ratio. Therefore, the main factor of $r^{A:U}$ will be the volume ratio, and the effects of the average AC ratio will be small. Thus, $r^{A:U}$ is approximately viewed as the volume ratio.

### Evaluation metrics of decomposed image quality

In addition to the diagnostic metric described above, two general evaluation metrics were employed in the experiments—the peak signal-to-noise ratio (PSNR) and Dice coefficient (DC)—for evaluating the absolute intensity and silhouette shape, respectively. To calculate DC, the decomposed image and ground truth (i.e., DRR generated from a CT image) were binarized. For the binarization threshold selection, we computed the average DC over the empirically determined threshold range of the intensity which was visually confirmed to obtain appropriate segmented regions of the target structures. To suppress the bias due to the threshold selection, we selected the threshold value for each method, by which the best average DC was attained. For statistical analysis, the Friedman test was employed to compare results for each group (in our case, the target structures were grouped into three: bones, hip muscles, and thigh muscles). The Wilcoxon test with Bonferroni correction for multiple comparisons was used.

## Experiments

### Data set

This study was approved by the Institutional Review Board at Osaka University (No.15056-3) and Nara Institute of Science and Technology (No.2019-M-6), and written informed consent was waived because of the retrospective design. All the following procedures were in accordance with the ethical standards as laid down in the 1964 Declaration of Helsinki and its later amendments or comparable ethical standards.

The summary of the data set used in this study is shown in Table 2. The data set consists of the lower extremity X-ray images of standing posture and the CT images of 475 patients (69 males and 406 females) acquired for the surgical planning of total hip arthroplasty at Osaka University Hospital, Suita, Japan. Ideally, the X-ray image and CT can be paired in a patient-wise manner, however, in practice, accurate 2D–3D registration of the muscles is quite difficult due to weak and incomplete edges of the muscles in X-ray images and nonrigid deformation with discontinuities between different muscles. Therefore, we used a CycleGAN-based framework of unpaired image translation.

**Figure 3:**
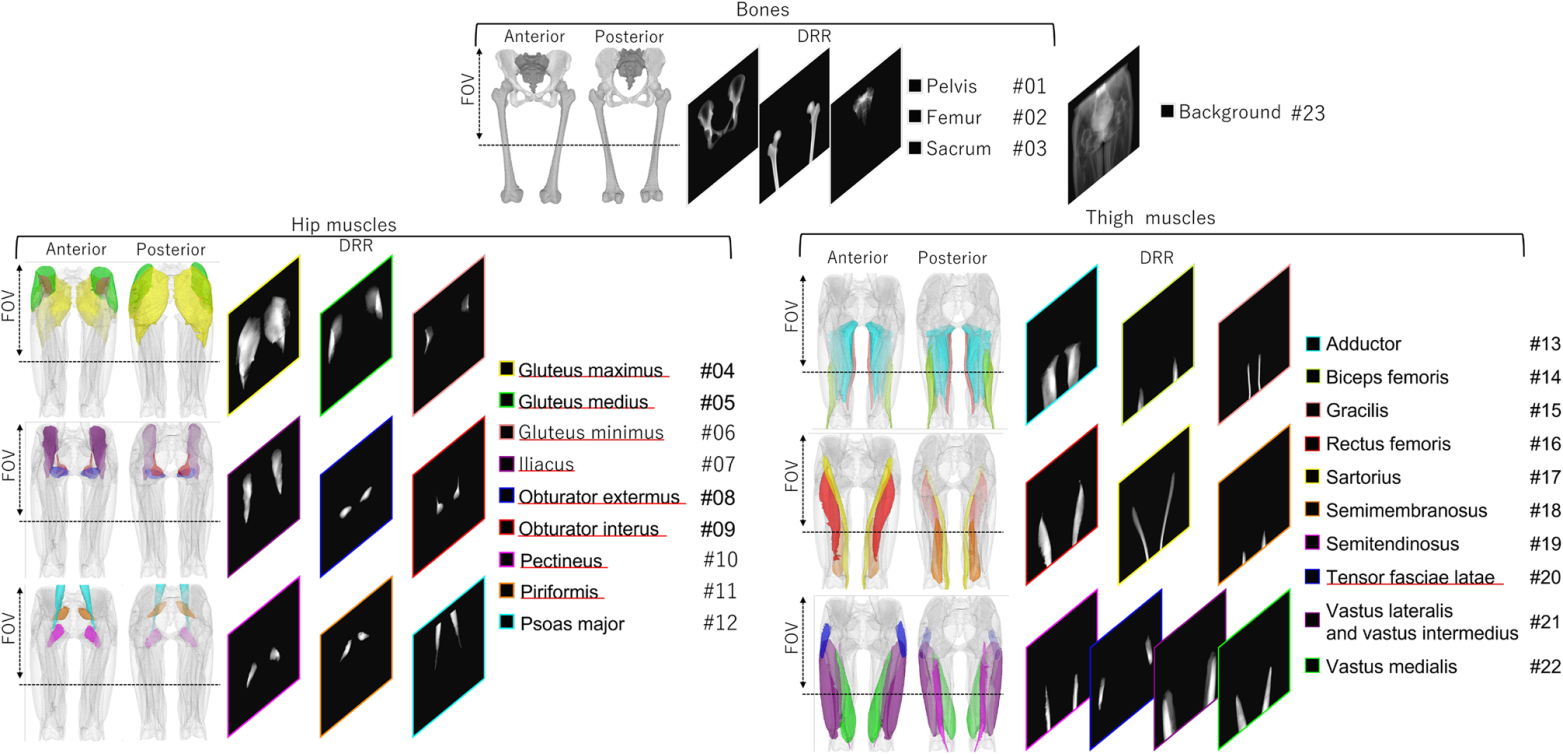
DRRs for three different bone regions (#01 $\sim$ #03), 19 different muscles (#04 $\sim$ #22) and the background (#23) created from CT images. The anterior/posterior 3D visualizations and DRRs of bones, hip muscles, and thigh muscles are shown for each region. The muscles whose entire regions were included within the FOV of the X-ray image were evaluated in the real image experiment. Red underlines indicate the evaluated muscles.

In order to construct the training data set, the CT images were automatically segmented with the Bayesian U-net^6^. The CT intensity in the unit of HU was first converted to a linear AC using the linear relationship assuming the monoenergetic X-ray. The muscle/bone regions segmented in this study were the 23 regions (3 bones, 19 muscles, and all other tissues as one object, which we call *background*) as shown in Fig. 3. The intensity values were normalized to [0,255] using the average of the maximum and minimum values of $I^{DRR}$ in the training cases. Since the normalization range was fixed throughout all cases, the decomposed DRRs synthesized by the proposed method can be denormalized to the original intensity range, which allows comparing the absolute density between cases. $I^{Xp}$ and $I^{DRR}$ were resized to $512 \times 512$ using the bi-cubic interpolation. The relatively small image size is due primarily to the CT resolution and GPU memory limitations. The voxel size of our CT was $0.7 \times 0.7 \times 1.0$ mm, as shown in Table 2, which limited the resolution of DRR. The reason for resizing the radiograph to 512 was to align it with the image size of the DRR (although we consider that it would be a possible future work to explore the efficient use of the high-resolution content in the radiograph by applying a super-resolution algorithm to DRRs).

**Table 2:** Summary of data set used in the experiments.

	Radiograph	CT
Image characteristics		
Posture	Standing	Supine
Image/volume and	$2900\times 2900$ (pixels)	$512\times 512\times 600$ (voxels)
Pixel/voxel sizes (approx.)	$0.14\times 0.14\ (\textrm{mm}^2)$	$0.7\times 0.7\times 1.0\ (\textrm{mm}^3)$

Sex	Male	Female
Patient demographics		
N (%)	75 (15.8)	400 (84.2)
Age (y.o.) mean ± std [IQR]	54.3 ± 17.2 [43.2, 66.0]	59.7 ± 13.1 [52.5, 69.0]
Height (cm) mean ± std [IQR]	166.9 ± 7.4 [161.1, 172.0]	154.4 ± 6.3 [150.0, 158.4]
Weight (kg) mean ± std [IQR]	67.9 ± 11.9 [61.0, 76.0]	54.9 ± 9.8 [48.0, 60.5]
BMI (kg/m$^{2}$) mean ± std [IQR]	24.3 ± 3.4 [21.9, 26.4]	23.0 ± 3.9 [20.2, 25.2]

### Experimental conditions

Two-fold cross-validation was performed in both simulation and real image experiments. The *patient-wise* split (237 and 238 patients) was performed. Twelve cases in the training dataset were employed for the validation.

The following four methods, including two conventional and two proposed methods summarized in Table 3, were compared. Conventional method #1 refers to the original CycleGAN^23^ without any additional features with generators of nine residual blocks and a discriminator of $142 \times 142$ PatchGAN^33^. Conventional method #2 refers to a method combining Conventional method #1 and the reconstruction loss proposed by Albarqouni et al.^22^ with a weight balance parameter of 0.5. Proposed method #1 used HL^27^ with the same generator as Conventional method #1. Proposed method #2 combined Proposed method #1 and the reconstruction GC loss. Regarding the reconstruction loss^22^, Li et al.^21^ and Zhang et al.^16^ noted that since it directly measures the difference between the virtual (i.e., DRR) and real X-ray images without taking into account their domain gap, it works only for a few real chest X-ray images with structures similar to the DRRs (i.e., when the domain gap is small). Therefore, the reconstruction loss^22^ was not included in the proposed methods. Detail of the parameters used in the experiments were summarized in Table 4

**Table 3:** Ablation study setup used in the experiments.

	Local enhancer in generator^27^	Reconstruction loss^22^	Reconstruction GC loss
Conventional #1	–	–	–
Conventional #2	–	$\checkmark$	–
Proposed #1	$\checkmark$	–	–
Proposed #2	$\checkmark$	–	$\checkmark$

**Table 4:** Summary of the parameters used in the experiments.

Types of parameter	Value	
Parameters common for all methods		
Optimizer	Adam	
Number of epochs	200	
Learning rate	0.0002 for the first 100 epochs and linearly decreasing to 0 for the later 100 epochs	
Number of generator’s residual block	9	
Patch size of discriminator	142$\times$142	

Method	Number of downsample layers in generator	Number of layers in discriminator
Parameters specific for each method		
Conventional #1	3	4
Conventional #2	3	4
Proposed #1	2	3
Proposed #2	2	3

### Simulation experiments

**Figure 4:**
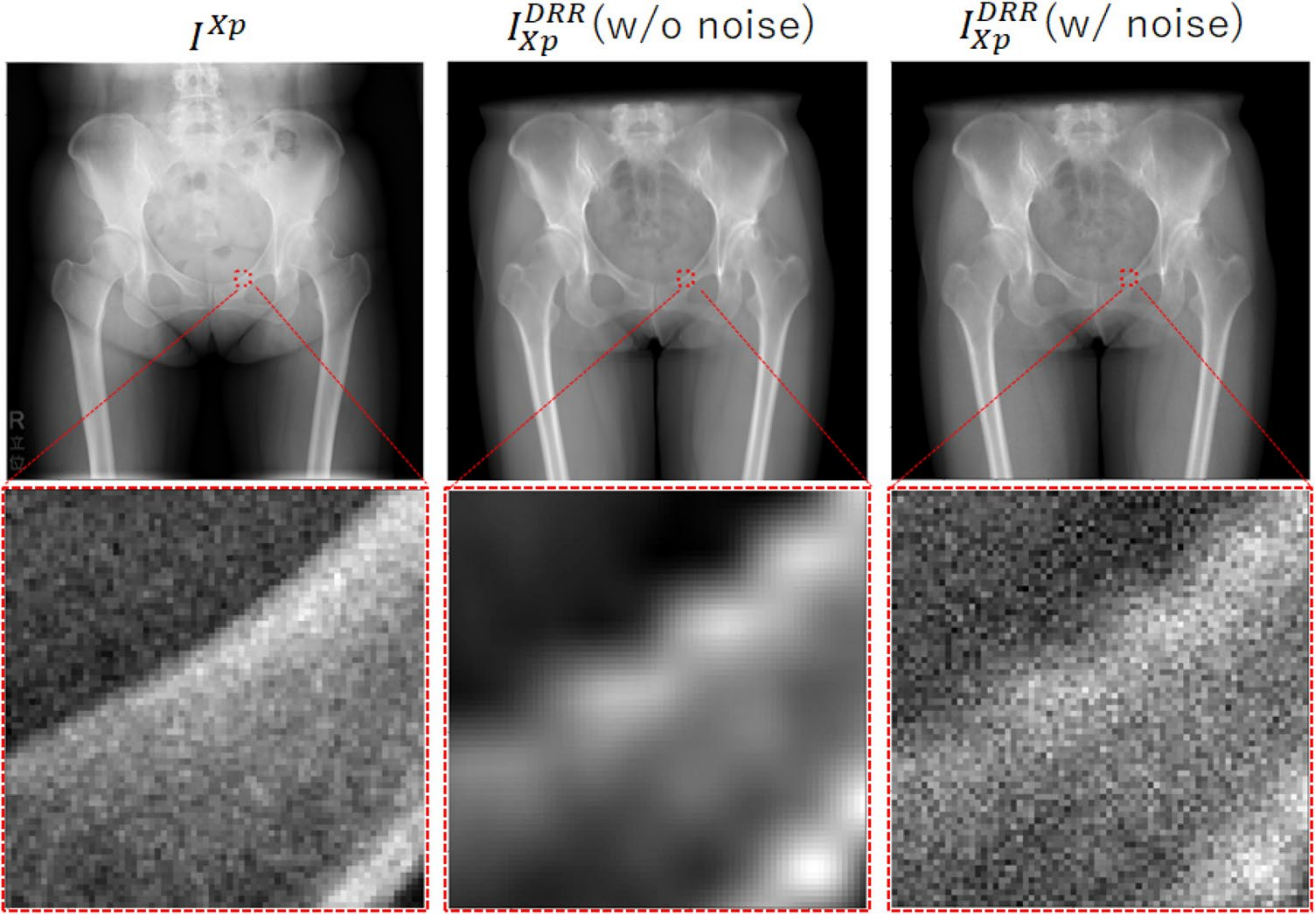
Comparison between real and simulated X-ray images. From left to right: Real X-ray image $I^{Xp}$, simulated X-ray image $I_{Xp}^{DRR}$ without noise, and $I_{Xp}^{DRR}$ with noise. The lower panel shows a magnified view of the red box in the upper panel, showing the similarity between the real X-ray image and $I_{Xp}^{DRR}$ with noise, which was used as input in the simulation experiments.

In the simulation experiments, input images were DRRs generated from CT images with additional Poisson noise, simulating the quantum noise appearing in real X-ray images (Fig. 4), which we denote by $I_{Xp}^{DRR}$. Projection parameters were set as described in “Projection model”.

### Real image experiments

Real X-ray images acquired with the actual clinical protocols were used. The evaluation was performed in two steps. (1) For rigid structures, namely pelvis and femur bones, PSNR and DC were evaluated using the DRRs obtained from the CT images registered to the X-ray images using a rigid 2D–3D registration^29^ as the ground truth. (2) For nonrigid structures, namely, muscles, the ground truth DRR (i.e., with the target muscles nonrigidly registered to that in real X-ray images) was difficult to obtain. Instead, the volume ratio of the (more) affected and (more) unaffected sides, introduced in “Muscle asymmetry metric”, was evaluated. An expert physician defined the affected side. We targeted nine muscles eliminating those not visible or only partially visible in the X-ray image’s field of view (FOV). Red underlines in Fig. 3 show the evaluated muscles.

**Figure 5:**
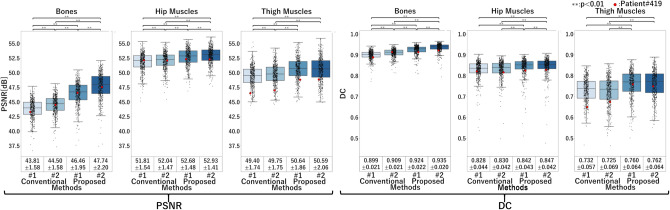
Results of decomposition accuracy in simulation experiments. Boxplots of PSNR and DC in the bone, hip muscle, and thigh muscle regions. One dot corresponds to one case (i.e., 475 data points for each boxplot). Boxes denote the first and third quartiles, and the median is marked with a horizontal line. The red dots correspond to the case shown in Fig. 6.

**Figure 6:**
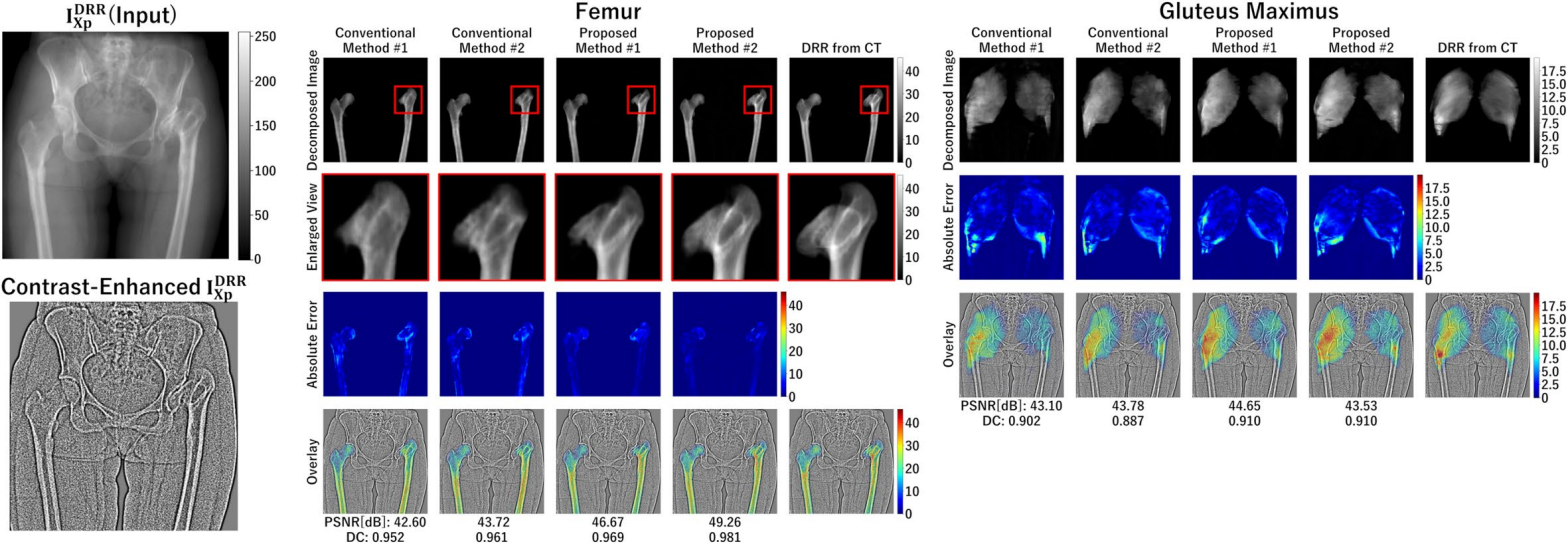
Illustrative decomposition results of simulation experiments. The visualized case (#419) corresponds to the red point in Fig. 5. The proximal femur, indicated by the red frame, is shown in the enlarged sub-window.

## Results

Table 5 shows the summary of the experimental results.

### Simulation experiments

The PSNR and DC for the four methods summarized in Table 3, using the simulated X-ray images (DRRs) $I_{Xp}^{DRR}$ (Fig.4 and “Simulation experiments”) generated by projecting the CT image, are shown in Fig. 5. The illustrative decomposition results are shown in Fig. 6. The accuracy of the decomposition results except for the background region was quantitatively evaluated. The muscle and bone regions were grouped into three groups—bone, hip muscle, and thigh muscle regions (Fig. 3), and PSNR and DC were averaged over each group for each case. The means and standard deviations of PSNR and DC of the four methods are shown in Fig. 5. Significant improvements were observed in Proposed methods #1 and #2 compared with Conventional methods #1 and #2 in both PSNR and DC. Further, Proposed method #2 showed significant improvements in PSNR and DC compared with Proposed method #1 except for thigh muscles.

As shown in Fig. 6, higher accuracy in Proposed method #2 was visually observed in the femoral head decomposition results, indicating that the combination of the HL and reconstruction GC loss in Proposed method #2 was especially effective in bone regions with higher contrast than muscles.

Please see Table S1 in Supplementary Materials for the complete ablation study where the three features (i.e., local enhancer in generator, reconstruction loss, and reconstruction GC loss) were excluded one at a time. The plot of PSNR as a function of the number of epochs in one example training trial is shown in Figure S1 in Supplementary Materials.

### Real image experiments

**Figure 7:**
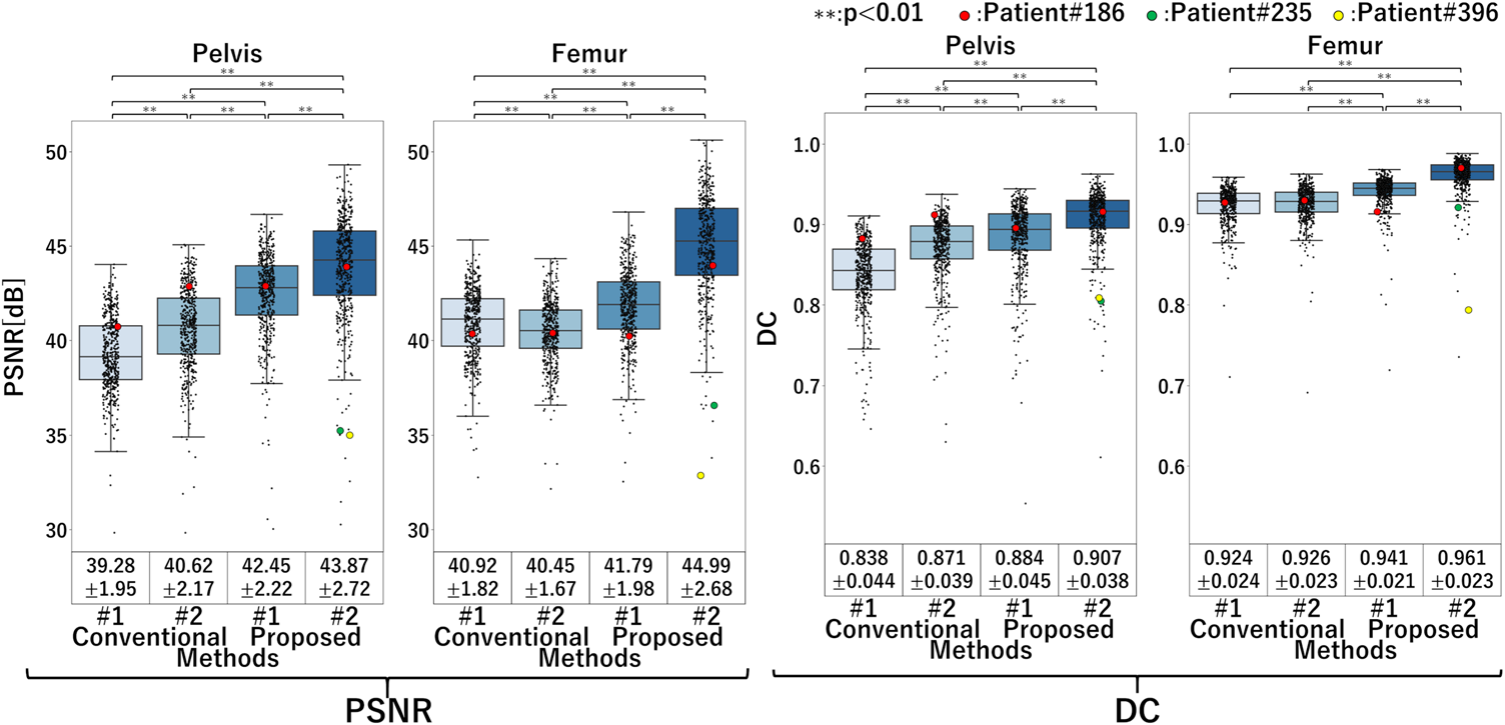
Quantitative evaluation of pelvis and femur in real image experiments. Boxplots of PSNR and DC. The red dots indicate the case (#186) shown in Fig. 9; the yellow and green dots indicate the representative failure cases (#235 and #396) shown in Fig. 10.

The quantitative evaluation results of the pelvis and femur regions decomposed from real X-ray images are shown in Fig. 7. The real image experiments also showed significant improvements in Proposed methods #1 and #2 compared with Conventional methods #1 and #2 in both PSNR and DC. Further, Proposed method #2 showed significant improvements in PSNR and DC compared with Proposed method #1 in both the pelvis and femur.

**Figure 8:**
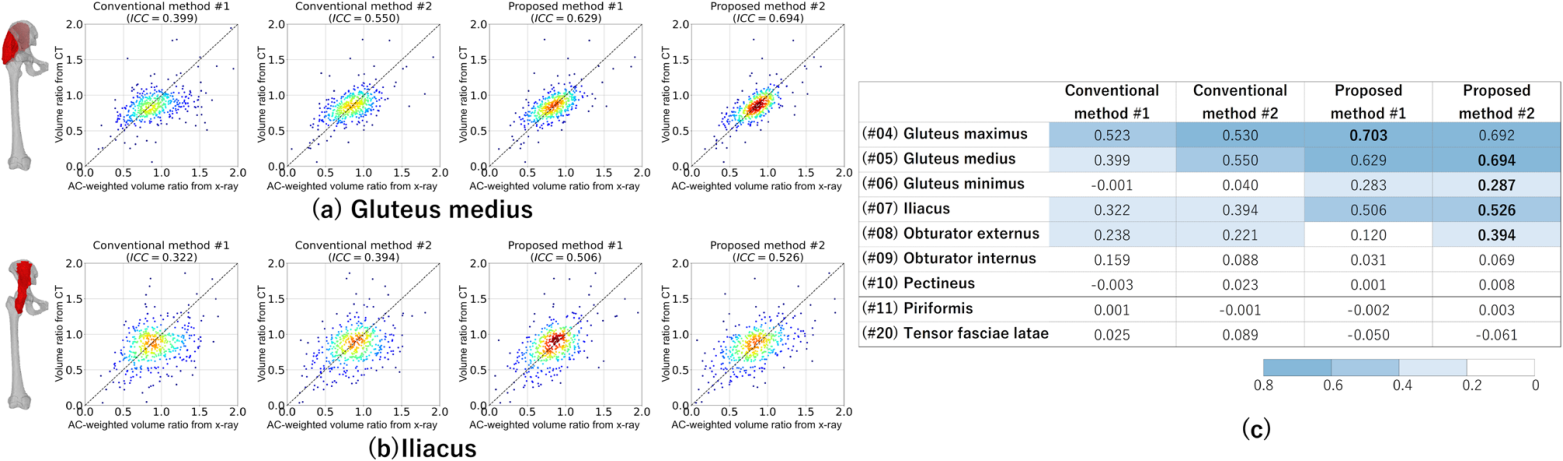
Correlation of the affected/unaffected volume ratio $r^{A:U}$ between muscles decomposed from real X-ray images and those calculated from CT images. (**a**,**b**) Scatter plots of four employed methods. (**c**) Intraclass correlation coefficients ($\rho$) for nine muscles analyzed in the experiments. The correlation coefficients were high for muscles with relatively large volumes, such as the gluteus maximus, gluteus medius, and iliacus muscles, especially in proposed method #2.

Figure 8 shows the comparison of affected/unaffected volume ratio $r^{A:U}$ (horizontal axis) measured from the real X-ray images of the nine evaluated muscles against the ground truth obtained from CT (vertical axis). Proposed method #2 tended to show a higher intraclass correlation coefficient (ICC) $\rho$^35^ for most muscles than other methods (Fig. 8c).

**Figure 9:**
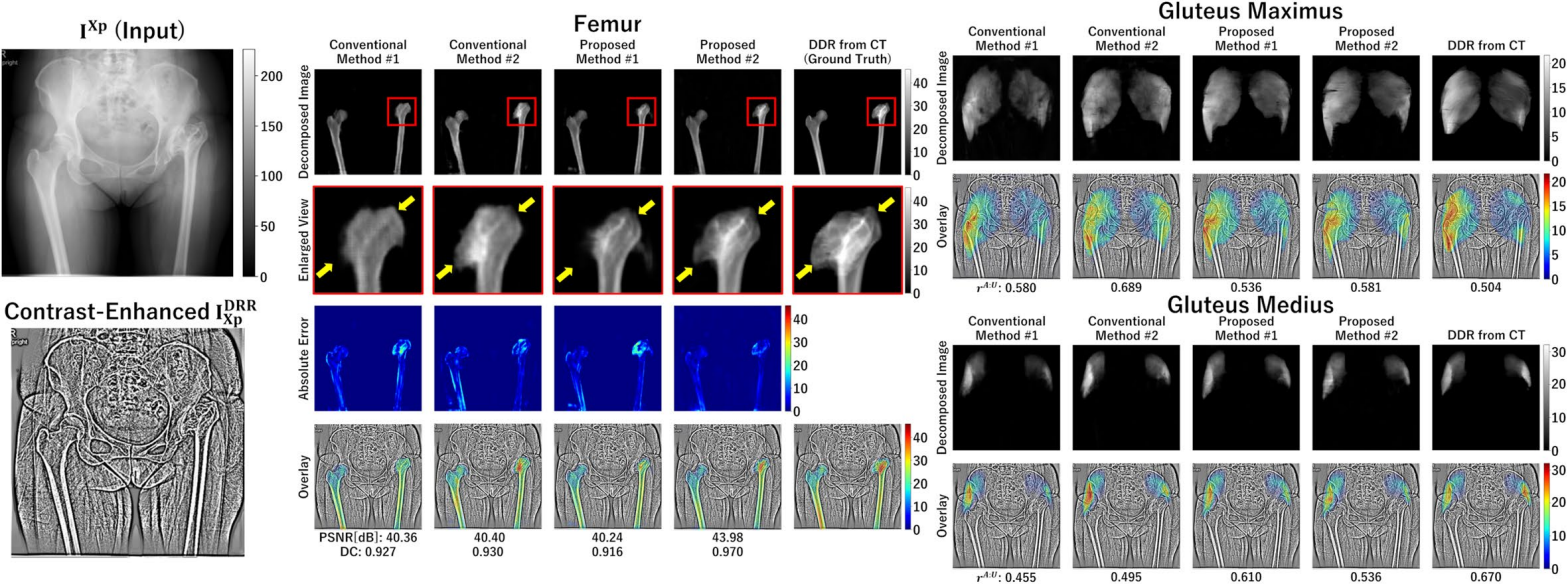
Representative results of the real image experiments. The femur and gluteus maximus muscles decomposed from the real X-ray image $I^{Xp}$ are shown.For the femur, the accurate ground truth was available via the 2D–3D registration, thus the heatmap indicating absolute error for each method is also shown, while for the gluteus maximus muscle, the DRR is not considered as the ground truth due to deformation between CT and X-ray image acquisitions. Thus, the DRR is shown as a reference, but the absolute error was not calculated. $r^{A:U}$ denotes the ratio of the affected/unaffected AC-weighted volumes defined by Eq. (9).

Figure 9 illustrates representative results for the femur and gluteus maximus muscles. Proposed method #2 demonstrated superior performance, especially in the proximal femur, indicated in the red rectangle, in delineating the structure boundaries (yellow arrows in Fig. 9) than the other methods.

**Table 5:** Summary of the experimental results.

Method	Bones		Hip muscles		Thigh muscles	
	PSNR	DC	PSNR	DC	PSNR	DC
Simulation experiments						
Conventional #1	43.81 ± 1.58	0.899 ± 0.021	51.81 ± 1.54	0.828 ± 0.044	49.40 ± 1.74	0.732 ± 0.057
Conventional #2	44.50 ± 1.58	0.909 ± 0.021	52.04 ± 1.47	0.830 ± 0.042	49.75 ± 1.75	0.725 ± 0.069
Proposed #1	46.46 ± 1.95	0.924 ± 0.022	52.68 ± 1.48	0.842 ± 0.043	50.64 ± 1.86	0.760 ± 0.064
Proposed #2	**47.74** ± **2.20**	**0.935** ± **0.020**	**52.93** ± **1.41**	**0.847** ± **0.042**	**50.59** ± **2.06**	**0.762** ± **0.064**

Method	Pelvis		Femur			
	PSNR	DC	PSNR	DC		
Real image experiments						
Conventional #1	39.28 ± 1.95	0.838 ± 0.044	40.92 ± 1.82	0.924 ± 0.024		
Conventional #2	40.62 ± 2.17	0.871 ± 0.039	40.45 ± 1.67	0.926 ± 0.023		
Proposed #1	42.45 ± 2.22	0.884 ± 0.045	41.79 ± 1.98	0.941 ± 0.021		
Proposed #2	**43.87** ± **2.72**	**0.907** ± **0.038**	**44.99** ± **2.68**	**0.961** ± **0.023**		

Highest values in each column are in [bold].

**Figure 10:**
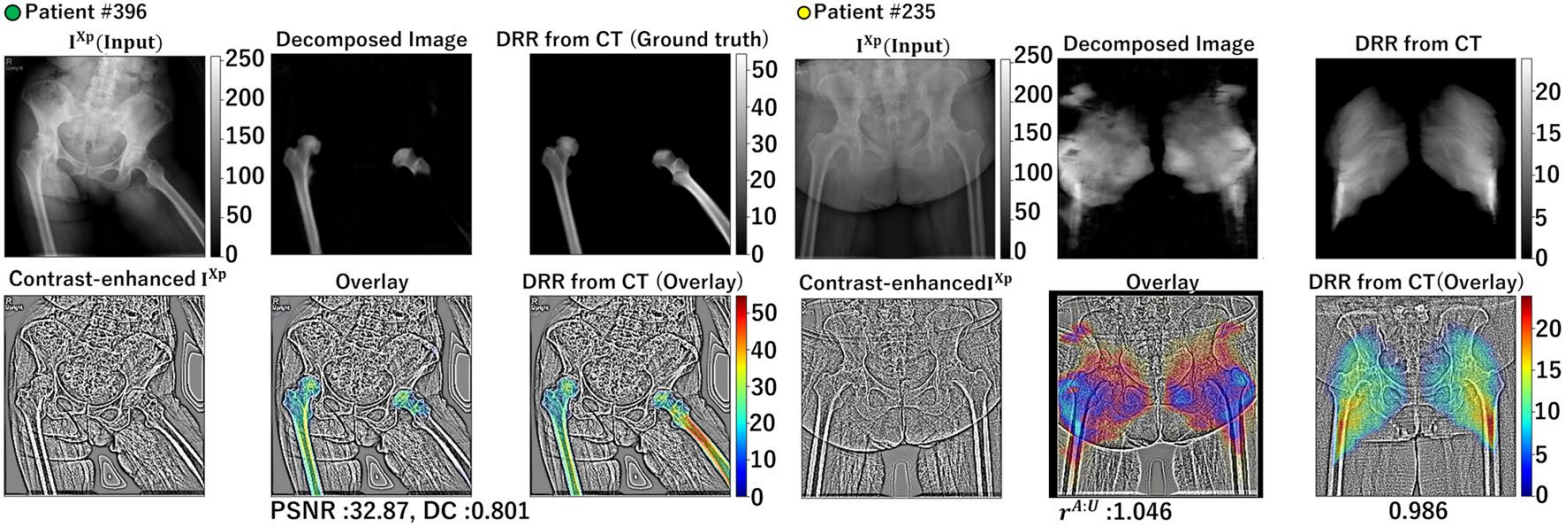
Representative failure cases decomposed from real X-ray image $I^{Xp}$. The first row shows $I^{Xp}$ with its contrast-enhanced images, the second row shows the decomposed images ${I}^{DRR}_n$, and the third row shows the overlay of the decomposed images onto the contrast-enhanced images. $r^{A:U}$ denotes the ratio of the affected/unaffected AC-weighted volumes defined by Eq. (9).

Figure 10 shows representative failure cases. The failure in patient #396 was attributed to the abnormal femur position, whereas that in patient #235 was attributed to excessive obesity compared with other patients in the training data set.

## Discussion

We presented methods to decompose an X-ray image of the hip into projection images each of which contains individual muscle and bone structures. We experimentally validated that the proposed CycleGAN-based image translation method with a combination of HL and reconstruction GC loss significantly improved the decomposition accuracy of 3 bones and 19 muscle structures compared with conventional methods. We showed that the quantitative decomposition achieved using the proposed methods, in which the intensity of the decomposed images has the unit equivalent to the line integral of the ACs, allowed evaluation of muscle asymmetry in case of relatively large muscles, which is clinically relevant in diagnosis and therapeutic planning in orthopedic surgery. To the best of our knowledge, this is the first peer-reviewed article that reports the quantitative validation of individual muscle decomposition from real X-ray images.

One advantage of the proposed methods is that automatically segmented 3D masks in CT were employed to construct the training dataset. We showed that automatic segmentation in 3D could be leveraged to construct a relatively large (475 cases) annotated training dataset without manual traces, which was used to train networks for decomposing individual muscles and bones from 2D X-ray images.

Our experiments evaluated the effects of each feature that we added to the original CycleGAN (Conventional method #1) independently. In the femur decomposition shown in Fig. 9, for example, the proximal femur region in the image decomposed by the original CycleGAN showed blurry and incorrect shapes at the femoral head and greater trochanter. By adding the reconstruction loss (Conventional method #2), the intensity patterns were better reconstructed but still blurry. The HL alone (Proposed method #1) provided a higher-resolution decomposition, removing the blurriness. By combining the reconstruction GC loss and the HL (Proposed method #2), the decomposed image better reproduced the intensity patterns, and the boundary of the greater trochanter became closer to the ground truth DRR, although the boundary of the femoral head, which is largely deformed due to osteoarthritis, was still not very close. Results shown in Figs. 7 and 9 validate the simulation experiments in Figs. 5 and 6 and support that the proposed methods, especially Proposed method #2 (incorporating the reconstruction GC loss and HL), are superior in the quantitative decomposition of the pelvis/femur regions in real X-ray images.

Our added features to CycleGAN were also shown to be effective for quantitative decomposition of individual muscles from real X-ray images. We demonstrated that the proposed methods showed optimal accuracy in estimating the affected/unaffected volume ratio of individual muscles (Fig. 8). Sufficiently high accuracy was obtained for large muscles such as gluteus maximus and gluteus medius; accuracy was not enough for small muscles. One contribution is that we demonstrated the potential usefulness of quantitative muscle volume analysis from plain X-ray images.

The two representative failure cases (Fig. 10) that showed low PSNR and DC in the pelvis and femur regions (green and yellow dots in Fig. 7) illustrated the limitation of the learning-based approach, where the cases that were not within the training data distribution failed. One of the failures (Patient #396) is attributed to the over flexion of the hip joint, likely due to inability to stand upright because of the flexion contracture, whereas the other failure (Patient #235) was due to excessive obesity that created skin surface edges different from most training data. One potential technique to mitigate this type of failure is data augmentation. For this purpose, simulations of muscle deformation accompanying with joint motion^36^ and simulations of body weight changes^37^ would need to be incorporated in data augmentation, which is in our future work.

In computer-assisted diagnosis in general, inaccurate prediction of the decomposition in a clinical situation is an important consideration. Hiasa et al.^6^ proposed a practical uncertainty metric as a surrogate inaccuracy measure for muscle segmentation in CT, which clinicians can use in clinical diagnosis. A similar approach, we believe, can be used in our radiography-based decomposition. It could also be extended to a more advanced image translation algorithm such as an uncertainty-aware translation.

One alternative solution to the real X-ray image decomposition problem is to divide the pipeline into two consecutive neural networks similar to the one proposed by Li et al.^21^ in the chest X-ray application, where (1) translation from a real X-ray image to an X-ray image-like DRR is trained with unpaired data sets and then (2) translation from the DRR to decomposed DRRs is trained with paired data sets. Unlike our one-step approach that combines these two steps into one network, the advantage would be in the second step, which allows the data augmentation by adding arbitrary geometric transform to CT, making the model more robust to various rigid misalignment, in exchange for instability and accumulation of errors in the training process of two GANs. A detailed comparison of the two approaches regarding the accuracy, flexibility to data augmentation, and training stability is left as future work.

## Conclusions

Using a large number of automatically segmented CT images as a training dataset, this study demonstrated the capability of decomposing the intensity distribution of individual muscles and bones from a single X-ray image. Despite some failures due to large differences from the training sample distribution, this study indicated that the volume ratio between the left and right muscles can be quantified to intraclass correlation coefficients of 0.7 with the ground truth. These findings support future research into frequent monitoring of musculoskeletal health for early detection of sarcopenia and osteoporosis.

## Supplementary Information

Supplementary Information 1.

## Data Availability

The data sets used and analyzed during the current study are available from the corresponding author upon reasonable request.
