# Supplementary material for: Decomposition of musculoskeletal structures from radiographs using an improved CycleGAN framework
Source: Sci Rep. 2023 May 25;13:8482. doi: 10.1038/s41598-023-35075-x (PMC10213012; doi:10.1038/s41598-023-35075-x)
Supplement: Supplementary file 1 — Supplementary Information 1. [file 41598_2023_35075_MOESM1_ESM.pdf]

Supplementary Materials: Decomposition of Musculoskeletal Structures from Radiographs Using an Improved CycleGAN Framework

Table S1. Results of the full ablation study (LE, RL, and RGC indicate Local Enhancer in generator, Reconstruction Loss, and Reconstruction GC loss, respectively. See the main text for detail.)

Method				Bones		Hip muscles		Thigh muscles	
	LE	RL	RGC	PSNR	DC	PSNR	DC	PSNR	DC
#1	-	-	-	41.48±3.29	0.921±0.046	59.41±4.64	0.804±0.099	58.35±4.71	0.740±0.171
#2	-	✓	-	42.75±3.43	0.929±0.045	60.66±4.84	0.819±0.108	59.07±4.71	0.769±0.150
#3	-	-	✓	43.93±3.27	0.937±0.046	60.79±4.87	0.817±0.107	59.08±4.68	0.763±0.154
#4	-	✓	✓	44.25±3.31	0.939±0.043	60.53±4.67	0.805±0.123	59.04±4.74	0.748±0.170
#5	✓	-	-	43.67±3.41	0.939±0.044	61.48±4.95	0.838±0.094	59.68±4.79	0.769±0.162
#6	✓	✓	-	43.86±3.42	0.939±0.045	61.67±5.15	0.840±0.093	59.65±4.78	0.773±0.154
#7	✓	-	✓	**45.52±3.61**	**0.951±0.040**	**61.88±5.18**	**0.849±0.089**	59.96±4.84	**0.783±0.152**
#8	✓	✓	✓	45.31±3.58	0.949±0.043	61.82±4.98	0.814±0.141	**59.98±4.86**	0.777±0.156

Note: This ablation study was conducted separately from the experiments in the main text. Conventional #1, #2 and Proposed #1, #2 in the main text correspond to methods #1, #2, #5 and #7, respectively. Although they were implemented in the same environment, each values differ from those in the main text due to the instability of GAN training, especially in an unpaired setting. However, the overall trend stays consistent with the results in the main text.

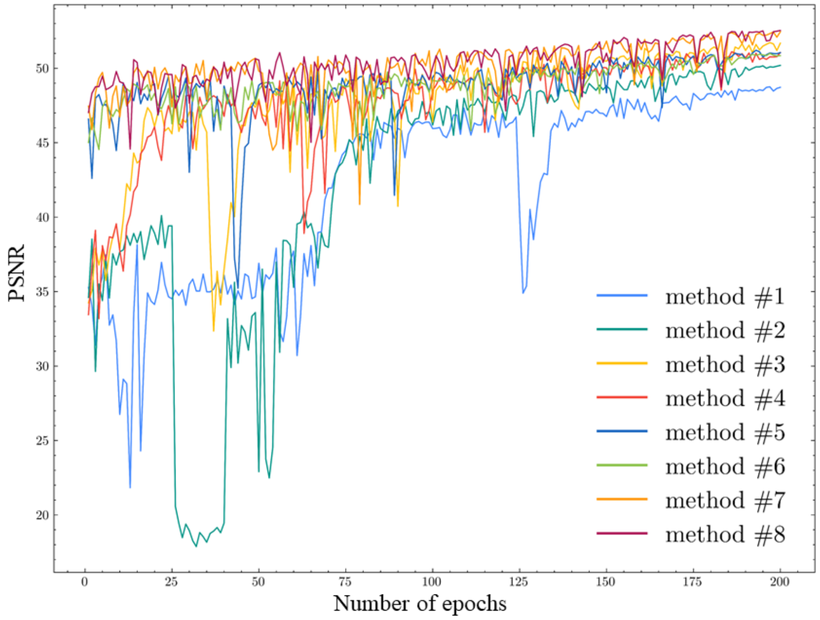

Figure S1. The plot of PSNR as a function of the number of epochs. Methods #1 and #2 move up and down unsteadily, indicating unstable behavior during the training, while methods #7 and #8 show a stable increase of PSNR. Note that the training curve changes at every training trial due to randomness in the initialization.
